# “It Comes in Steps and Stages”: Experiences of People Living with HIV in Achieving Employment

**DOI:** 10.3390/ijerph20186778

**Published:** 2023-09-18

**Authors:** Serena Rajabiun, Joseph S. Lightner, Marena Sullivan, Jessica Flaherty, Chau Nguyen, Joseph Ramirez-Forcier, Janet J. Myers

**Affiliations:** 1 Department of Public Health, University of Massachusetts—Lowell, Lowell, MA 01854, USA; 2 School of Nursing and Health Studies, University of Missouri-Kansas City, Kansas City, MO 64108, USA; lightnerj@umkc.edu; 3 Center for Innovation in Social Work and Health, School of Social Work, Boston University, Boston, MA 02215, USA; marenasullivan@gmail.com (M.S.); jessica.w.flaherty@gmail.com (J.F.); 4 HIV/AIDS Bureau, Health Resources and Services Administration, Rockville, MD 20852, USA; cnguyen1@hrsa.gov; 5 Positive Resource Center, San Francisco, CA 94103, USA; joe.ramirezforcier@prcsf.org; 6 School of Medicine, University of California—San Francisco, San Francisco, CA 94143, USA; janet.myers@ucsf.edu

**Keywords:** HIV, employment, navigation, interventions

## Abstract

People living with HIV who are seeking jobs experience unique barriers to obtaining employment at the individual, group, and community levels. Traditional employment assistance programs can provide support but may not be tailored to some people living with HIV who often experience barriers to work related to their social needs (such as housing instability) or their lack of consistent engagement in the workforce. To understand how people living with HIV return to work, in-depth interviews were conducted with 43 participants enrolled in interventions coordinating HIV care with housing and employment services at eight sites across the US. Four themes emerged on strategies to increase employment: (1) assessing and responding to employment needs that align with their socio-economic environment; (2) using social networks among family and friends for referrals and support; (3) engaging with navigators who are able to connect clients to skills building opportunities and job resources; and (4) addressing the system barriers such as helping with unmet basic needs (e.g. transportation), finding employers who can accommodate workers with income limits associated with public benefits, and helping immigrants, transgender individuals, and people experiencing homelessness secure legal documentsthat facilitate entry into employment by reducing stigmatized identities.

## 1. Introduction

Social determinants of health, such as poverty, homelessness, lack of education and unemployment, are associated with higher HIV prevalence and have been shown to impact HIV health outcomes [1]. There is evidence that programs providing support with housing assistance may be an effective structural intervention for people living with HIV because they not only improve housing stability, but also are associated with improved HIV outcomes [2,3,4,5,6]. Programs that included navigators as part of the care team to assist with housing and other social needs are associated with greater housing stability and improved HIV health outcomes for persons with HIV experiencing unstable housing with co-occurring mental health and substance use disorders [2,3]. Other studies have also found that providing rental assistance and housing placement services can be cost effective and promote HIV care engagement [4,5,6]. Among people living with HIV, in addition to stable housing, employment is also independently associated with being engaged in HIV testing, timely linkage to care, retention in care and medication adherence [7]. However, unemployment and housing instability often co-occur, further complicating associated barriers to HIV care and treatment.

Employment can increase financial self-sufficiency and reduce reliance on publicly funded benefits and other services, including health care, housing and supportive services currently provided by the Ryan White HIV/AIDS Program (RWHAP) and other federal programs. However, many HIV service delivery providers have not incorporated or coordinated employment services. Thus, more research is necessary to better understand the facilitators and barriers to employment in order to scale up effective supportive services for people living with HIV.

Recent studies demonstrate that some program models can address barriers and improve employment and other health outcomes in both persons experiencing unstable housing in HIV and non-HIV populations. One evaluation study of a housing and employment navigator model for families experiencing homelessness in three regions in Washington found a 7% higher rate of employment among participants who worked with a navigator specifically trained to support employment readiness, job skills and career resources [8]. Among people living with HIV, similar studies in Kansas City and New York found significant improvements in securing fulltime employment over time [9,10]. In these studies, a dedicated navigator/service coordinator proved effective in assessing employment skills, developing an individual employment plan with targeted goals, supporting employment readiness connection to job resources and educational training for clients. Navigators/coordinators also played important roles in communicating across various agencies to support a person experiencing unstable housing such as obtaining IDs, social security cards, and access to transportation [8,9,10].

With these promising models in mind, the Health Resources and Services Administration (HRSA), HIV/AIDS Bureau (HAB) RWHAP Special Projects of National Significance (SPNS) with support through the Secretary’s Minority HIV/AIDS Fund funded the initiative *Improving HIV Health Outcomes through the Coordination of Supportive Employment and Housing Services* (https://targethiv.org/housing-and-employment). The purpose of this initiative was to support the design, implementation, and evaluation of innovative interventions that coordinate HIV care and treatment, housing and employment services to improve HIV health outcomes for people living with HIV. Twelve demonstration sites partnered with local agencies with expertise in housing, healthcare, and/or workforce services to provide coordinated services to people living with HIV and to build local capacity for addressing social and medical needs in the community. Demonstration sites were funded to deliver innovative patient navigation models, tailored to their agency and community needs, and leveraged existing job centers and workforce supports, housing services and opportunities, and health care. Here, we report on the findings from in-depth interviews with clients who had received navigation, supportive housing and employment services. The goal of the study was to identify key factors facing clients seeking more permanent houisng and employment solutions and to understand how they were addressed by demonstration sites. Our hope is that by illuminating them in detail, key program elements will be adapted and replicated in the HIV health care system to improve employment outcomes and well-being for people living with HIV.

## 2. Materials and Methods

### 2.1. Study Design and Setting

This study was a sub-study of a larger multisite prospective, non-randomized study with a convenience sample of people living with HIV from 12 sites across eight states (CA (3), CT (1), GA (1), IL (1), MA (1), MO (1) NJ (1), NY (1), TX (2)) in the United States. All sites were located in urban areas with interventions based at four outpatient HIV clinics or federally qualified health centers, three city or county health departments, three AIDS Service Organizations, and two outpatient providers associated with hospital systems. All 12 sites received Ryan White Part A or Part B funding, with six sites also funded to provide comprehensive HIV medical care at the time of enrollment. In addition to RWHAP funding, eight sites or funded partners were either directly funded or partnered with a US Department of Housing & Urban Development (HUD) Housing Opportunities for People with AIDS (HOPWA) organization to provide housing or rental assistance to participants. One site had also subcontracted with a US Department of Labor-funded agency to provide employment assistance to participants. Another site subcontracted with a local employment assistance/job training organization. Other sites established direct referrals and/or onsite relationships with housing and employment agencies.

### 2.2. Intervention

Common elements of the interventions included: (1) using navigators as part of the care team to support clients seeking housing, employment and other social services, and (2) facilitating an integrated system-level coordinated delivery model with formal meetings between housing, employment and medical providers to share and exchange information and support referrals. Three sites hired 1–3 people to serve a caseload of clients and provide direct support with connecting individuals to employment, housing and health care services. The remaining sites used a team of specialized navigators (employment, housing case managers, and HIV case managers) who were both co-located and worked jointly with clients to address their housing, health care and employment needs. Despite the model, all staff completed HUD’s training program to support people living with HIV on seeking employment or returning to work [11,12].

Within these organizational contexts, navigators provided practical services, such as assisting clients secure IDs and legal documentation needed to work and obtain services, job search, resume services, housing application and search services, transportation services and referrals and linkages to HIV medical and behavioral health services. Navigators were an essential member of the care team often working collaboratively with the traditional HIV medical case manager.

### 2.3. Recruitment and Consent

We purposively selected participants based on three criteria: (1) enrolled in the demonstration site intervention and multi-site evaluation for a minimum of 6 months; (2) achieved a specific employment outcome, defined as gaining a job (part-time/full-time/paid with a direct payment or under the table/unpaid), or participating in job training, and (3) obtained more stable housing (defined as longer term transitional housing or permanent supportive housing) in the post intervention period. Eight demonstration sites participated in the sub-study and, after receiving training from the EC, were responsible for recruiting eligible clients for interviews and conducting interviews in English and Spanish.

### 2.4. Data Collection

**Figure 1:**
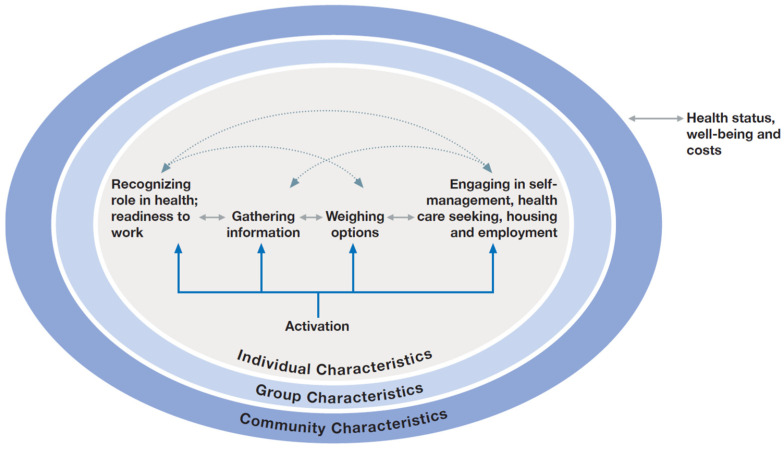
Adaptation of Mittler’s Consumer Engagement Framework to the Role of HRSA/SPNS Interventions to Examine Employment & Health Outcomes.

Our study and semi-structured interview guide was based on Mittler’s *Engaging Consumers in Health and Health Care in Communities* (ECHC) framework [13] which focused on individual, group and community level-factors affecting client experience with the intervention (Figure 1). At the individual level, we collected information related to participants’ socio-demographic characteristics, housing and employment status, level of food security, physical and mental health functioning, social isolation, unmet basic needs, length of time living with HIV and viral suppression prior to the intervention. This information was collected prior to the interventions’ implementation in baseline interviews and through medical chart data abstraction. Open-ended questions were asked to assess factors that facilitated employment. To understand how group-level factors influenced the outcomes of interest, participants were asked about who and what influenced their access to and use of information and material resources related to employment, with specific probes about the role of the intervention. Finally, information was gathered on community-level factors that could influence the operating environment of the intervention, such as the availability of housing, employment, transportation, and services that can ultimately affect an individual’s ability to be retained in care and virally suppressed. A copy of the question guide is included in Supplementary Materials Table S1.

Interviews were conducted by evaluation staff at the local site who participated in an initiative-wide qualitative interviewing training to standardize how the data were collected. Audio recordings and notes of interviews were uploaded to a secure server and de-identified. Interviews were transcribed and Spanish language interviews were translated into English for analysis. All but one participant consented to being audio recorded. Staff interviewers took notes on this client’s responses. Between November 2019 and March 2020, forty three (43) participants were interviewed with 5–8 clients per site. Participants received a $25 gift card upon completion of the interview. The study protocol was approved by Boston University Charles River Campus Institutional Review Board.

### 2.5. Data Analysis

Transcripts were reviewed for accuracy and imported into NVivo Version 12, for data management and to facilitate the coding process [14]. A codebook was developed based on Mittler’s ECHC framework and standard thematic content analysis techniques were used to categorize emerging findings [15,16]. Passages were deductively coded and analyzed for: individual factors, such as knowledge, attitudes or behaviors that helped or hindered employment seeking behavior; group factors such as having someone who helped a person seek or obtain employment and how the person supported their employment needs, and community factors such as larger organizational or structural issues that affected employment seeking behavior. The multisite evaluation team began with a pilot coding round in which four coders (two teams, two coders per team) reviewed the same transcripts. The four coders (M.S., M.C.S, J.K.D., J.F.) assessed inter coder reliability and reached consensus through discussion of each transcript. Areas of disagreement were resolved among the coders, with input from the project principal investigator. During this process, the coders also discussed emerging concepts and updated the codebook to incorporate additional codes derived from the data. Once the codebook was finalized, all transcripts were double coded. Study team members (M.S., M.C.S, J.K.D., J.F, SR, JM, CN, JMR) then reviewed the content of each code to develop a set of key themes mapped to the levels of the ECHC framework.

## 3. Results

Table 1 describes the participant characteristics prior to receiving intervention services. The majority of participants were male, working age adults (between ages 18–54 years) and identified as Black/African American or Hispanic. The majority described their sexual identity as LGBTQ+. Most had education beyond a high school diploma or GED. The average length of time living with HIV was 9.7 years and two-thirds of parpticipants were virally suppressed. Physical and mental health function score were on average 42.9 (13.2) and 38.4 (14.1) respectively, which indicates that our sample was facing more significant health challenges, relative to the general population. Approximately half indicated they were “homeless” prior to the start of the intervention and two thirds were unemployed. Approximately 14% received Social Security Income/Disability Income (SSI/SSDI). An overwhelming majority (93%) indicated they were experiencing “low/very low”significant food security. Nearly 70% had a history of incarceration. Participants reported an average, four unmet basic needs.

Four general themes emerged as facilitatators to achieving employment or other income generating activities among our participants, who were all unstably housed. From a participant perspective, interventions were most effective when they supported clients with skills and resources to find employment tailored to clients needs and interests. At a group level, interventions that supported the use of social networks, such as talking with family and friends to find employment or had a navigator facilitate these networks or make employent connections were critical to achieving employment success. Finally, participants said that interventions that addressed the structural barriers to employment, such as providing consistent transporation to work or obtaining an ID to apply for positions were the most helpful to them. Below we describe the key elements at the client, group and community level to impeding or supporting employment for people living with HIV.

### 3.1. Aligning Client Social Needs and Interests for Employment with the Realities of the Socio-Economic Environment

Participant employment needs were diverse. Some clients had experience with formal employment and long-term careers while others reported never fully engaging in formal employment and expressed a need for job trainings and education to re-enter the workforce since being diagnosed with HIV. Many clients reported their employment experience as being transitory; they were in and out of different jobs intermittent with periods of unemployment.

Given the range of experience, many clients reported difficulty in finding a position that was compatible with their current health status, their life histories (includng their incarceration histories), and their current skills and interests. Common reasons for inability to find a compatible job were due to history of incarceration, balancing work with other aspects of life with HIV, and having mental or physical health issues that could trigger events that precluded consistent work. Participants described the demands of available jobs, such as employers required too many hours or a mismatch of job duties to their skills. This lack of fit often contributed to work-related stress.

*I worked at the hotel for a month or two. It didn’t work out. It was a lot of stress. I had this job where I worked six days a week, nine hours a day. So… So, I went, probably a month without having a job, which was super rough. And then I got hired into this nursing home job. And it was good, you know… [But] my issue with this job was [that] it was extremely, extremely stressful when I was there.*

For other clients who had struggled with substance use or mental health disorders in addition to housing instability, the stress and worry related to a job contributed to their concern of a return to problematic substance use or mental health issues. One client described how pushing themself to hold a job affected their ability to manage their substance use and mental health:

*Before I moved into the sober living and the temporary housing I [had been] was couch surfing … I was working at the gym and I had this little side job …and I was doing recovery and it kinda pushed me over the edge a little bit, and uh… I was doing too much… I relapsed at that point*

#### 3.1.1. Having Knowledge about the Employment Process, Legal Requirements and How to Seek and Apply for Work

Another barrier for multiple participants was understanding the process of obtaining a job because they had not worked in the US before or because many jobs were only listed online. While this limited knowledge did not impact participant’s readiness to work, it did require a higher degree of self-motivation because they also had to gain new to be able to be responsive to opportunities.

*Sometimes, I go to an area where I know there’s a lot, and just walk around. Most of the time they’re, like, Apply online! Apply online!”…What I do is, I take a notepad and I’ll go… Pizza Hut… Pizza Hut is online. Then you may go to a Mom-and-Pop shop, where they’re, like, “we can interview you right now! Can you sit down with us?” So, they’re less formal. It depends on where you go.*

#### 3.1.2. Supporting Their Family and Themselves with More Than Just Basic Needs

Despite challenges, many participants described motivational factors for seeking and obtaining employment. For example, they wanted to work since it allowed them to afford more thanlife necessities and also gave them the opportunity to better take care of family members.

*I have a daughter. It’s money. It’s an income. It’s the ability to pay things my way, pay the rent. It’s not having gone month to month worrying about tightening my belt. Choosing… you know. We’re gonna have to help her, we’re only gonna eat such and such, this, this not. We’re gonna have to buy the cheapest, smallest, lowest quality meat to make it stretch, you know, for five meals. That’s the reason why I went back to work. To provide with the basics for my daughter and being comfortable doing that…not having to struggle for the basics.*

In addition to satisfying basics needs, other participants reported wanting employment to afford goods and participate in activities that gave them satisfaction and would improve their life.

*You know how much it costs to furnish your house? And I like cute stuff! In order to get that cute stuff you gotta have cute money and got a cute job… everything gotta be cute but, just like with any other thing you use this as a stepping stone to get something better. I’m happy I got a roof over my head, I can cook and eat, and do whatever I need to do on a daily basis, but when I get right, right… don’t wanna be right, right now, I wanna be right, right, right! If you wanna get better, you gotta strive to get better.*

Participants also expressed a motivation to work because they had a desire to return or maintain the position and career path of their choice because it was a vocation.

*I am going to be persistent to be a teacher because this is my area. I am teacher. Maybe people say: what? you can do whatever you want! But not what I really want. I’m a teacher, I want to teach children because this is my area. This is my career. So, far that reason, persisting all the time.*

### 3.2. Using Social Networks of Family and Friends and via Navigators

While self-motivation factors at the client level contributed to obtaining employment, group factors such as the ability to leverage people in their social networks were a key factor to finding and obtaining employment. Social networks were described as two types: (i) having a circle of family and friends and (ii) consisting of people who were there to help with employment, such as thenavigator or the intervention staff. Participants described family and friends as a source for building their self- esteem that so they can work and that HIV was not something that affected their ability to work. Also family and friends were sources of job referrals and helping participants with completing applications.

### 3.3. The Role of the Navigator in Supporting Employment Opportunities

In the absence of family and friends, the navigators employed by SPNS program were frequently identified as the main source of their social support with obtaining and maintaining employment.For many participants the navigator provided the role of building their self-esteem in the belief they could work, connecting them to the resources, taking the time to building their skills such as creating a resume, completing a job application, or obtaining an ID their first step they could apply for a job.

#### 3.3.1. Building Confidence and Self-Esteem to Pursue Employment

Some clients also reported that encouragement from the navigators inspired them to pursue new employment opportunities: [Navigator] said to me is there anything you need I’ll help you, she’s with me no matter what decision I plan on making in the future, [Interventionist] she said she’s gonna be with me. The support that I have is more than I can ever ask for so. Similarly, another client said that with regards to their employment journey, their navigator as “… she just motivates me; she gives me hope”.

*Other clients described the navigator as inspiring them and building their self-esteem during the employment process: She is just patient, I get flustered and she just keeps telling me things to keep me real calm, it’s the voice that you need to hear, you know, so, every time I get [makes a noise as if angry], she was kind, you know, I got many thoughts going over my head and she listens to them… she kind of organizes my brain thoughts. Like, I said, she is like a big sister that doesn’t give up on you, you know?*

#### 3.3.2. Navigators Connect to Resources for Employment Opportunities

The navigators and their agencies provided access to computers to look for jobs and connection to job fairs thus helping clients directly connect to employers. As one client described:

*[Name of the navigator] was the one that sent me to the job fair. So, it was successful for me, because I had like four job offers. I never had that many job offers.*

#### 3.3.3. Build Skills for Obtaining a Job

Another role for the navigators was helping clients with completing job applications especially for positions that only accept via online, resume writing, conducting mock interviews, and link to outside job trainings. As one client said, “I feel like [the local intervention] gave me tools [for employment], and not just for the right now”. Another client described:

*[Name of Navigator] was a big help with looking for jobs. [Name of Navigator] helped me do my resume, helped me redo my resume, helped fill out applications online and stuff like that and she showed us how to look up jobs and tested us to how to interview when you go to interview for a job, she did all that one-on-one, you know, so, that was helpful.*

Finally, given that clients also may be unstably housed or experiencing homelessness, or due to their immigration status one of the first steps is getting documentation and legal IDs to successfully obtain employment.

*[I received] encouragement and support from [name of navigator], mostly. And help with my ID. [Name of navigator] understands what it’s like for me… helping me with the ID and all. I’ve had a lot of bad experiences with jobs and looking for jobs because of who I am, and [name of navigator] knows that and understands that. And that helps a lot”.*

### 3.4. Structural and Community Factors That Contribute to Employment for People Living with HIV

Many participants faced structural challenges in the path towards successful employment. These factors included finding employers who did not discriminate against them based on their transgender identity or immigration status; finding an employer who understood the need for accommodations due to health reasons; earning wages that supplemented their income and maintained their public benefits such as health insurance, housing or Social Security Income; and having access to reliable transportation to maintain employment. Others described a desire to work because they needed a higher wage to afford the cost of living in their area.

#### 3.4.1. Have an Accommodating Work Environment and Policies in Place to Support People Living with HIV

Clients described how employers failed to recognize their need for accommodations to maintain their health status. In some cases, employment instability was a result of the lack of understanding:

*I knew something huge was going on [with my health] So…I approached my employer and said, you know, I can’t qualify for FMLA, but I have done some research with ADA that I know that you have to honor mental health or, needing accommodation, so I asked for an accommodation, I actually qualified for an accommodation and was given two periods, twice a month, of two days, so that I could have a mental health [break] without it [having a] negative impact on me, but, at the same time, after going through this process, I’ve had enough no-call, no-shows that they ultimately let me go.*

#### 3.4.2. Access to Transportation for Obtaining or Maintaining Employment

Transportation frequently came up as a factor that influences employment. As one client described:

*“one of the hardest things is finding a great job, interviewing over the phone and being prepared to go to a physical interview, but then realizing that I am only going to get halfway there or two thirds the way there by bus and then I am going to have to Uber or walk a long distance”.*

#### 3.4.3. Need for Legal Documents & Approval of SSI/SSDI to Work

Other clients noted that it was a complicated and long process to obtain legal approval and documentation required for employment. Examples included Supplemental Security Income approval and obtaining copies of professional certificates and drivers licenses with changed sex markers. As one participant described their challenge with obtaining employment due to legal status:

*I am in the middle between doing interviews and applications. But this is a real good job, the program is, I don’t know what is going on with my immigration status, because my Green Card expired. So, this is a big problem I am working on that, I have my attorney help me. I pay it for myself…when I get a job I am going to be fantastic. I’m with problems with the immigration status, because immigration took a long time for getting my Green Card or my permission for getting a job. So, it depends on them.*

#### 3.4.4. Stigma and Discrimination Due to Identity

The lack of legal recourse and protections for transgender people or immigration status presented a challenge for continuous employment. In one case, the client faced discrimination based on having an identification card that did not reflect their current gender:

*I’m looking, but every time I go see someone about a job, it’s always a problem once I show my ID. Their ad says they have a job, but as soon as they see me and my ID, they tell me there ain’t no job………it’s been filled, whatever. It’s hard for people like me. {Interventionist] sent me to this lawyer and she was helping me. It’s taking a long time… I guess there’s a lot of people trying to change their ID, ‘cause she don’t call me back. I gotta follow-up again and see what’s happening. Anyway, I’ve had a lot of jobs and trouble always comes. It’s hard.*

## 4. Discussion

The National HIV Strategy calls for the need to address the social determinants, such as employment and housing, in order to improve HIV health outcomes and quality of life [1]. One of the key measures is improved employment as part of the quality of life. Our study findings contribute to these goals by identifying the multilevel challenges for people living with HIV in seeking and obtaining employment and potential solutions through interventions and policy changes.

Our findings are similar to other studies that have documented barriers and facilitators for people living with HIV from seeking and obtaining employment. Fear of impact on physical health, discrimination in the workplace and impact on receipt of public benefits were the key barriers to employment [7]. Other studies have indicated that anticipated stigma, defined as perception of poor treatment by community members, and poor mental and physical health and functioning were predictors of employment barriers [17]. Key facilitators having a supportive work environment, where supervisors are educated about HIV and offer accommodations, having a flexible work schedule to take time off for medical appointments, and having social support from health care providers, family and friends [7]. Our study contributes to the evidence by identifying the individual factors for motivation such as desire for a better life for themselves and family or returning to their vocation. In addition, our study found that the group factors, such as social networks with family and friends in the employment process and the role of external supports such as navigators in finding job training resources and learning the on line job search and application processes. These latter skills are critical to support people with a history of housing and employment instability.

Furthermore, our findings contribute to the evidence that navigators can assist people living with HIV in addressing the structural factors that impede seeking employment or returning to work, such as having legal identification, consistent transportation, and finding a job in a work friendly environment that promotes productivity and well-being and reduces stigma and discrimination. Other studies have doucment the roles that navigators play in in supporting people living with HIV who experience unstable housing with improving HIV health outcomes and housing stability [18,19]. Our study builds upon this evidence for navigators as part of the care team in assisting with employment stability.

Based on these findings, we highlight a number of key program and policy implications to support employment for people living with HIV. First, HIV and social service programs must develop comprehensive screening tools for staff to assess client readiness, motivation for employment, barriers associated with social determinants, such as housing and poverty, and then integrate employment goals into existing care plans. Addressing social disparities that limit employment opportunties is critical. For some people living with HIV, identifying perceptions and worries about seeking or returning to work and their abilities in navigating the employment sector is also a first step. For people living with HIV out of the workforce for a while, building skills and comfort with technology and applying and searching for jobs online may be critical and instilling confidence they can find gainful employment. Furthermore, Assessment tools should be specific to identify needs related to type of work, hours, location to address transportation needs.

Second, the use of navigators or training existing case manager as part of the team to connect people living with HIV to relevant job fairs, training opportunities to enhance skills or enroll in social media programs such as LinkedIn or online job search programs. Navigators can also receive training on how to ask questions that identify needs and tools that calculate for clients the amount of additional income that can be gained without affected public benefits. This task can help clients weigh their options between forgoing benefits to have a higher paying position to fulfill career aspirations or selecting more limited employment and wages to maintain their benefit status. In our study, participants obtained work because of their intrinsic desire to work or they were inspired by a navigator’s encouragement in seeking and maintaining work. Finally, navigators can also play a specific role in assisting client to address barriers creating from stigma and discrimination by obtaining new IDS, name changes on social security cards, connect to legal services, and documentation status for formal employment.

Third, testing interventions, such as the use of peer support groups to enhance motivation and share employment seeking strategies and their effect on quality of life and HIV health outcomes is critical. Several of the SPNS programs used peer navigators. In addition, organizations and clinics can establish partnerships with agency that specialize in employment to assess and match desired employment needs.

At a structural level, policy changes are needed to address the persistent barriers that contribute to returning to work or seeking employment for people living with HIV, most of which are due to social disparities faced by this vulnerable group. Our current ADA laws support accommodations for people living with HIV. However, many employers lack awareness or be unwilling to support accommodations necessary for time off to care for physical and mental health status. Ryan White and other social service providers can work with organizations to create friendly policies allowing for flexible work hours or working from home, when necessary. In addition, having a system that supports client readiness to work with training and orientation on workplace policies and procedures and technological skills, provides opportunities for professional development and pathways to promotion are critical. At a federal level, changes to the rules and raising the income limits at which reductions or stopping benefits are necessary to encourage and motivate people living with HIV to obtain and maintain employment.

Our findings also indicate the need for greater investment in resources to address barriers for specific populations who experience unstable housing, such as those who are lack legal documentation to work in the US and persons of transgender and homeless experience. One of the key first steps to obtaining employment is having an identification card with one’s legal name. The study was not designed nor probe in depth about the specific role of gender identity, legal documentation and impacts on employment. Future studies could explore the specific needs of the LGBTQ+ population and other persons with undocumented status to tailor interventions and policies that support their stable employment.

Our study has a number of limitations. This was a convenience sample of people living with HIV who participated in housing and employment interventions and, therefore, lacks external validity to other populations with HIV. However, given that this was a national sample, collected across a wide geographic region, and serving people living with HIV all experiencing initial housing and employment instability, these results represent perspectives of people living with HIV suffering from housing and employment challenges nationally. The findings provide rich insight to personal experience and highlight the need to strengthen policies and programs to support employment stability. Further studies are needed to replicate the findings for specific population groups. Second, the study lacks internal validity since the types of employment models and support with navigators did not follow standard protocol. Our study findings provide evidence for further testing interventions that can improve employment and health outcomes among people living with HIV.

## 5. Conclusions

Seeking employment or returning to work can be a challenging for people living with HIV who are burdened by navigating a fragmented system for their housing, employment and health-related needs. Individual, group, structural, and community factors all impact an individual’s readiness and ability to obtain and sustain employment. Future research should continue to advance interventions focused on reducing social disparities and tigma and improve access to employment and training opportunities for people living with HIV. Creating more flexible work environments and policies that align with their life histories, goals, interests and physical and mental health and well-being are critical components to increasing employment opportunities for people living with HIV.

## Figures and Tables

**Table 1:** Characteristics of Participants Across 8 Study Sites at Baseline (n = 43).

	n	%
**Age (Years)**		
Mean (SD)	39 (12)	
Range	20–63	
≤30	13	30.3
31–54	25	58.1
≥55	5	11.6
**Sex**		
Male	28	65.1
Female	10	23.3
Transgender/or Other	5	11.6
**Race/Ethnicity**		
Non-Hispanic White	3	7.0
Non-Hispanic Black	25	58.1
Hispanic	10	23.3
Other (includes multiracial)	5	11.6
**Sexual Orientation**		
Heterosexual	19	44.2
Lesbian/Gay/Homosexual	19	44.2
Bisexual	3	7.0
Other	2	4.7
**Education**		
Less than high school	8	18.6
High School	12	27.9
Beyond high school	23	53.5
**Place of Origin**		
North America (Not U.S)	3	7.0
South America	2	4.7
Africa	1	2.3
1 of the 50 states in the U.S	37	86.0
**Housing Status**		
Literally homeless	22	51.2
Imminent risk of losing housing	4	9.3
Unstably housed/at risk of losing housing	17	39.5
**Employed**		
Yes	15	34.9
No	28	65.1
**Received Supplemental Security Income**		
Yes	6	14.0
No	37	86.0
**Food Security**		
High or marginal food security	3	7.0
Low food security	15	34.9
Very low food security	25	58.1
**Number of Unmet Service Needs** **(Max 13)**		
Mean (SD)	4 (2)	
Range	0–8	
**Previously incarcerated**		
Yes	30	69.8
No	13	30.2
**Clinical characteristics**		
**How long has the participant lived with HIV, years (n = 40)**		
Mean (SD)	9.7 (8.6)	
Range	0–31	
**Viral Suppression (n = 37)**		
Not virally suppressed or >200 viral load	9	20.9
Virally suppressed or ≤200 viral load	28	65.1
**Quality of Life measures**		
**Physical Component Score**		
Mean (SD)	42.93 (13.15)	
Range	6.16–61.8	
**Mental Component Score**		
Mean (SD)	38.4 (14.14)	
Range	10.43–61.78	

## Data Availability

The data presented in the study are available on request from the corresponding author. The data are not publicly available due to restrictions due to privacy.

## Supplementary Materials

The following supporting information can be downloaded at: https://www.mdpi.com/article/10.3390/ijerph20186778/s1, Table S1: Question guide for Clients in HRSA/SPNS Housing & Employment Project.
