# Supplementary material for: “It Comes in Steps and Stages”: Experiences of People Living with HIV in Achieving Employment
Source: Int J Environ Res Public Health. 2023 Sep 18;20(18):6778. doi: 10.3390/ijerph20186778 (PMC10531408; doi:10.3390/ijerph20186778)
Supplement: Supplementary file 1 [file ijerph-20-06778-s001.zip › ijerph-2375396-supplementary.pdf]

Supplementary Table 1: Question guide for Clients in HRSA/SPNS Housing & Employment Project

Question: Please describe what has helped you with finding employment and/or job training in the [Program Name] program?

1. Can you tell me about your current employment situation?
   PROBE: Are you currently working or do you expect to work soon?
   PROBE: What type of job or job training are you doing?
2. What was it like working with [Interventionist Name] in getting a job/job training?
   PROBE: What were some of the most helpful things?
3. What was different about your experience working with [Interventionist Name] compared to your previous experiences looking for a job/job training?
4. What made you want to find a job/job training?
   PROBE: Is there anything about the [Program Name] program that encouraged you to find a job/job training?
5. What other supports did you have to help you find a job/job training?
   PROBE: Did any family or friends help you?
   PROBE: Were there other resources or community programs/groups that helped you?
   PROBE: Was there anything about the physical area or your local neighborhood that was a support to you?
6. Did getting a job/job training affect your housing or encourage you to look for new housing?
   IF YES: Can you tell me about that?
